# Effects of moxibustion aroma stimulation and lower-limb warm stimulation on blood pressure and cerebral hemodynamics: a pilot randomized trial

**DOI:** 10.3389/fphys.2026.1898938

**Published:** 2026-09-08

**Authors:** Xiaohan Li, Qiaofeng Lin, Pan Zeng, Shuang Gong, Junjie Bian, Lingling Wu, Weijun Sun, Kun Wei, Min Tang

**Affiliations:** 1 Department of Neurological Rehabilitation, Ningbo Rehabilitation Hospital, Ningbo, China; 2 Ningbo Key Laboratory of Brain Cognitive Rehabilitation, Ningbo Rehabilitation Hospital, Ningbo, China; 3 Hydrotherapy Center, Ningbo Rehabilitation Hospital, Ningbo, China

**Keywords:** blood pressure, cerebral hemodynamics, functional near-infrared spectroscopy (fNIRS), lower-limb warm stimulation, moxibustion aroma

## Abstract

**Objective:**

Sensory and thermal interventions may acutely affect both peripheral cardiovascular regulation and cortical activity, but their comparative effects remain unclear. This study investigated the acute effects of moxibustion aroma stimulation, lower-limb warm stimulation, and their combination on blood pressure (BP), heart rate (HR), and cortical hemodynamics in healthy young adult men.

**Methods:**

In this pilot randomized trial, 40 healthy young adult men were randomly assigned to a resting control group, moxibustion aroma stimulation group, lower-limb warm stimulation group, or combined stimulation group (n = 10 each). Each intervention lasted 20 min. Systolic BP (SBP), diastolic BP (DBP), and HR were measured at 0, 5, 10, 15, and 20 min. Functional near-infrared spectroscopy (fNIRS) was used to assess oxygenated hemoglobin (HbO) responses and functional connectivity (FC) in the bilateral prefrontal and motor cortices.

**Results:**

Significant time effects were observed for SBP, DBP, and HR (all P < 0.001), together with significant group × time interactions for SBP (P < 0.001), DBP (P = 0.011), and HR (P = 0.037); the overall group effects were not significant. Significant group × stage interactions were observed for HbO in all four ROIs and for all interregional FC measures (all P < 0.001). Late-stage reductions in BP and HR were associated with increases in HbO in selected cortical regions (r = 0.577–0.820, all P < 0.001).

**Conclusion:**

Moxibustion aroma stimulation, lower-limb warm stimulation, and combined stimulation were associated with distinct time-dependent patterns of cardiovascular and cortical hemodynamic responses in healthy young adult men. The associations between late-stage reductions in BP/HR and regional increases in HbO suggest concurrent peripheral cardiovascular and cortical hemodynamic changes during stimulation. However, these preliminary findings do not establish a causal central–peripheral regulatory mechanism or demonstrate the superiority of any intervention.

## 1 Introduction

Blood pressure (BP) regulation is a fundamental component of cardiovascular homeostasis and depends on the dynamic interaction between peripheral vascular mechanisms, autonomic nervous system activity, and central neural control (Dampney, 2016; Matić et al., 2026). Increasing evidence suggests that cortical and subcortical networks, particularly prefrontal regions, are involved in autonomic cardiovascular regulation, linking higher-order neural processes to peripheral physiological responses (Thayer et al., 2012; Shantsila, 2016). In this context, non-pharmacological interventions capable of modulating both peripheral and central regulation have attracted growing attention in integrative medicine.

Moxibustion is a traditional therapeutic modality widely used in East Asian medicine (Kim et al., 2011; Park et al., 2020). Previous studies have mainly focused on its thermal, local circulatory, and clinical effects (Li et al., 2019; Park et al., 2020; Nakahara et al., 2022). However, burning moxa also produces a characteristic odor that may serve as a biologically meaningful sensory stimulus. Olfactory input projects to limbic and paralimbic structures involved in emotional processing, arousal, and autonomic regulation (Soudry et al., 2011; Thompson and Umphred, 2019), suggesting that moxibustion aroma may influence cardiovascular and cerebral responses even without direct thermal contact. Nevertheless, the independent physiological effects of moxibustion aroma remain poorly characterized, and few studies have simultaneously assessed its peripheral cardiovascular and cortical hemodynamic responses.

Lower-limb warm stimulation is another simple and clinically relevant non-pharmacological intervention (Thomas et al., 2017). Previous studies have shown that warm-water immersion and passive heat exposure can promote peripheral vasodilation, alter blood-flow distribution, improve vascular function, and reduce BP in humans (Bailey et al., 2016; Brunt et al., 2016a; Thomas et al., 2016; Cheng, 2023). In addition to these peripheral effects, sustained thermal and somatosensory input from the lower limbs may engage cortical regions involved in sensory integration, autonomic adjustment, and motor-related processing (Fukuie et al., 2019). However, most previous studies have focused primarily on peripheral hemodynamic outcomes, and the accompanying cortical responses remain insufficiently understood. Moreover, direct comparisons among moxibustion aroma stimulation, lower-limb warm stimulation, and their combination are limited.

Functional near-infrared spectroscopy (fNIRS) enables non-invasive, real-time monitoring of oxygenated hemoglobin (HbO) responses and functional connectivity (FC) in superficial cortical regions, including the prefrontal and motor cortices (Ferrari and Quaresima, 2012). Therefore, fNIRS provides a suitable approach for examining whether acute peripheral cardiovascular changes during sensory and thermal stimulation are accompanied by cortical hemodynamic responses. Because sex-related differences in autonomic cardiovascular regulation, thermoregulatory responses, olfactory sensitivity, and cerebral hemodynamics may increase physiological variability, the present pilot study included only healthy young adult men to improve internal consistency and reduce potential confounding related to sex differences and hormonal fluctuations (Koenig and Thayer, 2016; Inagaki et al., 2021; Caeiro et al., 2023).

It remains unclear whether moxibustion aroma stimulation, lower-limb warm stimulation, and their combination produce distinct acute cardiovascular and cortical hemodynamic responses when evaluated within the same experimental framework. To address this knowledge gap, the present four-arm pilot randomized trial included a resting control group and directly compared the three stimulation conditions during a standardized 20-min intervention. BP, heart rate (HR), ROI-level HbO responses, and interregional FC were assessed simultaneously. By integrating peripheral cardiovascular and cortical hemodynamic measurements, the study aimed to provide preliminary evidence on coordinated physiological responses to olfactory and thermal/somatosensory stimulation. Specifically, we aimed to characterize the time-dependent cardiovascular and cortical hemodynamic responses across the four groups and to examine associations between late-stage reductions in BP/HR and cortical HbO changes. We hypothesized that the temporal trajectories of cardiovascular and cortical hemodynamic responses would differ between the resting control group and the three stimulation groups.

## 2 Methods

### 2.1 Study design, setting, ethics, and trial registration

This was a single-center, four-arm, parallel-group pilot randomized trial conducted at Ningbo Rehabilitation Hospital in Ningbo, China, between January and March 2026. All 40 participants, including the 10 participants assigned to the resting control group, were recruited and tested concurrently during this study period. The resting control group was part of the original four-group randomization procedure and was not recruited as a subsequent or non-concurrent comparison group. The study comprised a 5-min resting baseline period followed by a single 20-min intervention period. Cardiovascular and fNIRS measurements were obtained before and during the intervention. Only acute physiological responses were assessed; no post-intervention or long-term follow-up assessments were performed.

The study protocol was approved by the Institutional Review Board of Ningbo Rehabilitation Hospital (Approval No. 2026-KY-11) and was conducted in accordance with the Declaration of Helsinki. All participants were informed of the study purpose, procedures, and potential discomfort before enrollment, and written informed consent was obtained from each participant before data collection.

The study was not prospectively registered. It was initially conducted as an exploratory physiological pilot study. The lack of prospective registration is acknowledged as a methodological limitation. This study is reported in accordance with the CONSORT 2025 statement, and the completed reporting checklist is provided as **Supplementary Table 1**.

### 2.2 Participants, recruitment, and sample size

Forty healthy adult men were recruited at Ningbo Rehabilitation Hospital using volunteer sampling, a non-probability sampling approach. This approach was adopted because the study was designed as an exploratory pilot study and participant recruitment was based on feasibility and available resources. Randomization was applied only after enrollment for allocation to the four study groups and did not refer to participant sampling from the source population.

The inclusion criteria were as follows: (a) male sex and age ≥ 18 years; (b) self-reported good health, with no history of cardiovascular, neurological, psychiatric, or major musculoskeletal disorders; (c) no known olfactory dysfunction, allergy, or intolerance to moxibustion smoke or odor; and (d) no skin lesions, infection, sensory disturbance, or other contraindications to lower-limb warm-water immersion.

The exclusion criteria were as follows: (a) unstable BP or a history of clinically significant cardiovascular disease; (b) use of medications or consumption of alcohol, caffeine, nicotine, or other substances that could substantially affect cardiovascular or cerebral hemodynamic measurements within 12 h before testing; and (c) inability to remain seated quietly or cooperate with fNIRS recording.

Healthy participants were selected because this exploratory pilot study was designed to characterize acute physiological responses to the stimulation paradigms while minimizing potential confounding from cardiovascular, neurological, psychiatric, or other clinical conditions. Only male participants were included to reduce potential physiological variability related to sex differences and hormonal fluctuations and to improve the internal consistency of this preliminary sample.

No formal *a priori* sample-size calculation was performed. Given the exploratory nature of this pilot study, a pragmatic sample of 40 participants (10 per group) was selected on the basis of recruitment feasibility and available resources to characterize preliminary cardiovascular and cortical hemodynamic response patterns. As shown in **Table 1**, the four groups did not differ significantly in age, height, weight, systolic BP (SBP), diastolic BP (DBP), or HR at baseline.

**Table 1:** Baseline characteristics of the four groups.

Variable	Resting control (n = 10)	Moxibustion aroma stimulation (n = 10)	Lower-limb warm stimulation (n = 10)	Combined stimulation (n = 10)	F	P
Age (years)	23.0 ± 1.3	22.0 ± 2.0	23.1 ± 1.9	22.3 ± 1.1	1.091	0.365
Height (cm)	168.9 ± 8.6	169.6 ± 9.1	168.7 ± 7.3	169.2 ± 6.3	0.025	0.995
Weight (kg)	62.4 ± 8.1	59.3 ± 7.1	59.7 ± 9.9	64.9 ± 7.1	1.030	0.391
SBP (mmHg)	116.4 ± 3.9	119.7 ± 5.0	116.4 ± 7.6	118.0 ± 6.2	0.728	0.542
DBP (mmHg)	74.5 ± 3.4	79.3 ± 6.5	77.9 ± 8.3	76.4 ± 6.7	1.005	0.402
HR (beats/min)	72.0 ± 3.7	79.3 ± 9.0	77.4 ± 14.0	75.9 ± 13.0	0.832	0.485

Values are presented as mean ± SD. SBP, systolic blood pressure; DBP, diastolic blood pressure; HR, heart rate.

### 2.3 Randomization, allocation concealment, and blinding

Participants were randomly allocated in a 1:1:1:1 ratio to the resting control, moxibustion aroma stimulation, lower-limb warm stimulation, or combined stimulation group using a random allocation procedure implemented by drawing lots. Before participant enrollment began, 40 identical opaque, sealed allocation bags were prepared for the four prespecified groups, with 10 bags assigned to each group. After enrollment, each participant selected one bag without replacement to determine group allocation. The bags remained sealed until selection, thereby maintaining allocation concealment. The allocation bags were prepared by X.L.; participants were screened and enrolled by Q.L.; and group assignment and intervention implementation were conducted by P.Z. No blinding was implemented. Participants, intervention providers, outcome assessors, and data analysts were aware of group allocation because the moxibustion aroma and warm-water interventions were readily perceptible.

### 2.4 Experimental protocol and interventions

All experiments were conducted in a quiet, temperature-controlled laboratory. Participants were instructed to remain seated comfortably throughout the experiment, avoid talking, and minimize head and body movements during fNIRS recording. Before the intervention, the fNIRS probes were positioned, signal quality was checked, and participants remained seated quietly for a 5-min resting baseline period.

**Figure 1:**
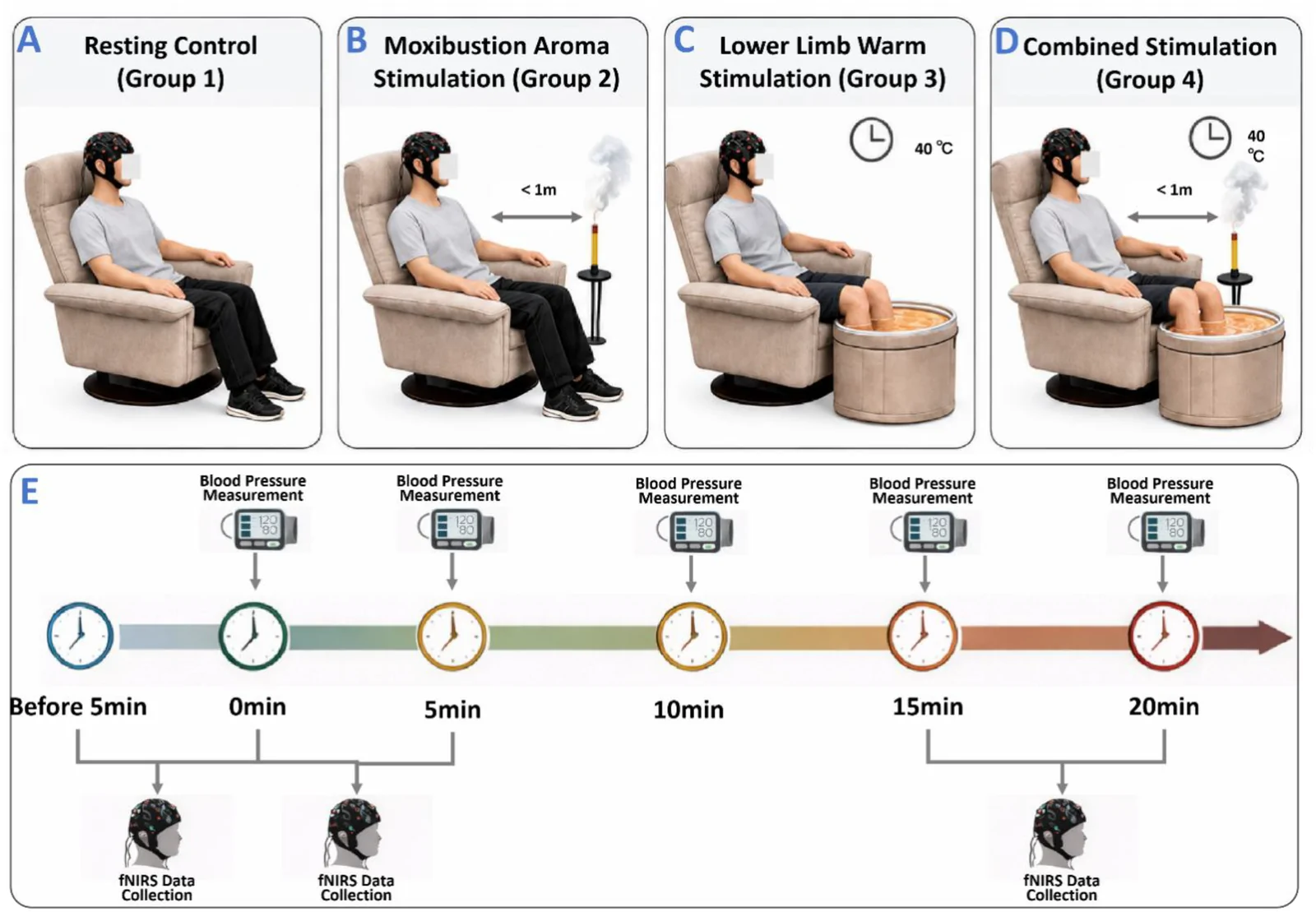
Schematic diagram of the experimental protocol. **(A)** resting control; **(B)** moxibustion aroma stimulation; **(C)** lower-limb warm stimulation; **(D)** combined stimulation; **(E)** experimental timeline.

In the resting control group (Group 1), participants remained seated quietly for 20 min without receiving aroma or thermal stimulation (**Figure 1A**).

In the moxibustion aroma stimulation group (Group 2), participants remained seated while being exposed to the odor of a lit moxibustion stick positioned approximately 1 m in front of them (**Figure 1B**). The approximately 1-m distance was selected to ensure continuous perception of the moxibustion aroma while minimizing direct thermal stimulation from the burning moxa, thereby allowing the sensory effects of the aroma to be examined with minimal contribution from local heating.

In the lower-limb warm stimulation group (Group 3), participants remained seated with both lower limbs immersed below the knees in a thermally insulated foot bath maintained at 40 °C (**Figure 1C**). The water temperature of 40°C was selected because previous human studies have shown that hot-water immersion at approximately 40–42°C can elicit measurable peripheral vascular and cardiovascular responses while remaining feasible for controlled short-term exposure (Miwa et al., 1994; Brunt et al., 2016a; Thomas et al., 2017).

In the combined stimulation group (Group 4), participants simultaneously received moxibustion aroma stimulation at a distance of approximately 1 m and lower-limb warm stimulation at 40°C (**Figure 1D**).

The total intervention duration was 20 min in all four groups. A 20-min intervention period was selected because previous studies have demonstrated measurable cardiovascular and hemodynamic responses within this time frame during thermal or moxibustion-related stimulation (Li et al., 2010; Maruyama et al., 2010; Vyas et al., 2020). BP and HR were measured at five time points: immediately before the intervention (0 min) and at 5, 10, 15, and 20 min during the intervention. Measurements at 5-min intervals were used to characterize the temporal pattern of cardiovascular responses throughout the intervention. fNIRS data were collected during three predefined stages: the 5-min resting baseline stage before the intervention, the early intervention stage (0–5 min), and the late intervention stage (15–20 min) (**Figure 1E**). The early and late intervention periods were predefined to represent the initial and late phases of stimulation, respectively, allowing comparison of cortical hemodynamic responses at different stages of the intervention.

All interventions and measurements followed standardized procedures to ensure consistency in participant posture, moxibustion distance, warm-water immersion temperature, and environmental conditions throughout the experiment.

### 2.5 Outcome measures and data collection

#### 2.5.1 Blood pressure and heart rate measurements

SBP, DBP, and HR were measured using an Omron electronic sphygmomanometer (Omron Healthcare Co., Ltd., Kyoto, Japan) under standardized seated conditions. Measurements were obtained from the right upper arm using an appropriately sized cuff positioned approximately at heart level. Participants were instructed to remain relaxed, avoid talking, and minimize unnecessary movement during measurement. The same device, arm, cuff-placement procedure, and measurement protocol were used throughout the study.

#### 2.5.2 fNIRS data acquisition

Cerebral hemodynamic responses were recorded using a multichannel fNIRS system (ETG-4100, Hitachi Medical Corporation, Tokyo, Japan). The system emitted near-infrared light at wavelengths of 695 and 830 nm. The emitter-detector distance was 3 cm, and the sampling interval was 0.1 s, corresponding to a sampling rate of 10 Hz.

**Figure 2:**
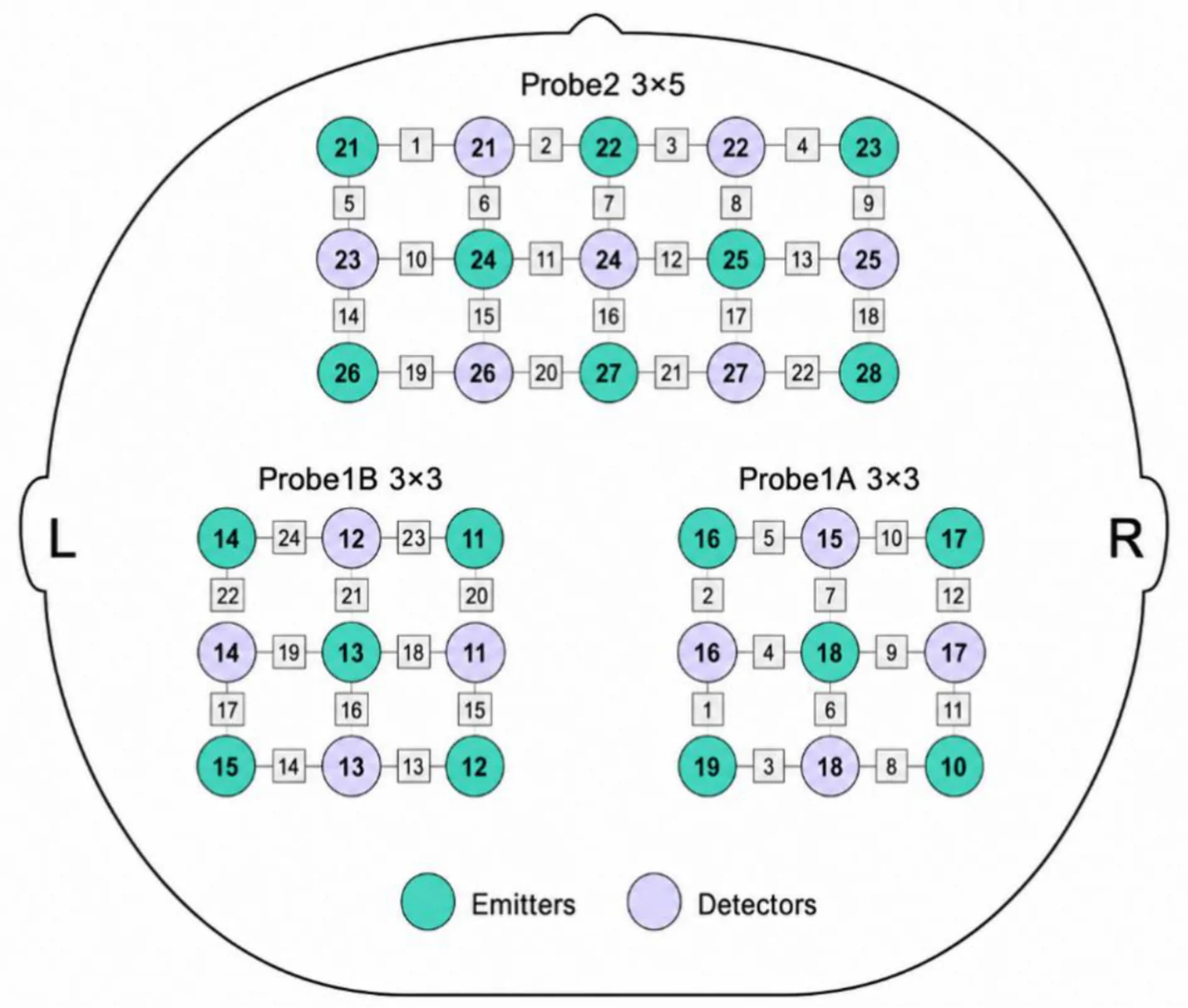
Schematic diagram of the fNIRS probe configuration, emitter–detector arrangement, and channel distribution.

The probe configuration consisted of 18 emitters and 15 detectors, forming a total of 46 measurement channels. Three probe sets were applied: one 3 × 5 array covering the bilateral prefrontal cortex and two 3 × 3 arrays covering the left and right motor cortices. A schematic diagram of the fNIRS probe configuration, emitter-detector arrangement, and channel distribution is shown in **Figure 2**. According to the probe arrangement, channels were assigned to four regions of interest (ROIs): the left prefrontal cortex (LPFC), right prefrontal cortex (RPFC), left motor cortex (LMC), and right motor cortex (RMC). Before data acquisition, probe placement and signal quality were checked to ensure stable optode-scalp contact throughout the recording.

### 2.6 fNIRS data processing and analysis

The fNIRS data were exported from the ETG-4100 system and processed offline in Python using NumPy, Pandas, SciPy, and Matplotlib. Oxygenated hemoglobin (HbO) was selected as the primary hemodynamic measure because it is generally considered sensitive to stimulation-related changes in cortical hemodynamics (Ferrari and Quaresima, 2012; Scholkmann et al., 2014; Pinti et al., 2020).

Before preprocessing, all channels were screened for signal quality. Poor-quality channels were identified according to predefined qualitative criteria, including obvious signal loss, long flat segments, abrupt non-physiological spikes, marked baseline instability, or excessive noise throughout the recording (Brigadoi et al., 2014; Pollonini et al., 2016). No fixed quantitative threshold was applied for channel rejection because the original preprocessing procedure did not use an automated signal-quality metric. Channel quality was visually confirmed using the raw HbO time series. Channels meeting the poor-quality criteria were excluded from subsequent ROI-level averaging, whereas channels with transient artifacts but acceptable overall signal quality were retained after detrending and filtering. A total of 48 channels were excluded across all participants, corresponding to approximately 2.6% of the 1,840 channel observations (40 participants × 46 channels). The excluded channels comprised 8 in the LPFC, 10 in the RPFC, 16 in the LMC, and 14 in the RMC, with 0–2 channels excluded per participant. Although slightly more exclusions occurred in the motor cortical ROIs, excluded channels were distributed across all four ROIs rather than being concentrated in a single region. No participant was excluded because of excessive channel loss. Given the low overall exclusion rate (2.6%) and the distribution of excluded channels across all four ROIs, channel loss was considered unlikely to substantially influence the ROI-level analysis.

To reduce slow baseline drift and physiological noise, the HbO time series were linearly detrended and band-pass filtered at 0.01–0.20 Hz (Scholkmann et al., 2014; Santosa et al., 2018). This frequency range was selected to preserve slow hemodynamic fluctuations related to the prolonged stimulation paradigm while reducing very slow drift and high-frequency physiological interference. The filtered data from the three predefined recording stages—baseline, early intervention (0–5 min), and late intervention (15–20 min)—were then used for subsequent analysis.

For each participant, ROI-level HbO values were calculated by averaging the available valid channels within each ROI during each stage. If a channel was excluded because of poor signal quality, it was not included in the ROI average. Baseline-corrected ROI-level HbO changes, defined as ΔHbO, were calculated as the mean HbO value during each intervention stage minus the mean HbO value during the baseline stage.

FC was assessed using ROI-level HbO time series. Pearson correlation coefficients were calculated between each pair of ROIs within each stage, including LPFC–RPFC, LPFC–LMC, LPFC–RMC, RPFC–LMC, RPFC–RMC, and LMC–RMC. The resulting correlation coefficients were converted to Fisher’s z values before statistical analysis to improve normality.

### 2.7 Statistical analysis

Statistical analysis were performed using IBM SPSS Statistics (IBM Corp., Armonk, NY, USA). Continuous variables are presented as mean ± standard deviation (SD). Baseline characteristics were compared among the four groups using one-way analysis of variance (ANOVA).

Mixed-design repeated-measures ANOVA was used to evaluate SBP, DBP, HR, ROI-level HbO, and ROI-to-ROI FC. Group was treated as the between-subject factor. For SBP, DBP, and HR, time was the within-subject factor, with five levels: 0, 5, 10, 15, and 20 min. For ROI-level HbO and FC, stage was the within-subject factor, with three levels: baseline, early intervention (0–5 min), and late intervention (15–20 min). The main effects of group and time/stage and the corresponding group × time/stage interactions were evaluated. Greenhouse–Geisser-corrected results were reported when the sphericity assumption was violated. Bonferroni-adjusted *post hoc* comparisons were performed, where appropriate, to examine within-group changes across time or stages and between-group differences at individual time points or stages.

Reductions in BP and HR were calculated relative to the pre-intervention values at 0 min. Specifically, ΔSBP, ΔDBP, and ΔHR were calculated as the value at 0 min minus the value at each subsequent time point, with positive values indicating reductions from the pre-intervention level. Baseline-corrected ΔHbO was calculated as the mean HbO value during each intervention stage minus the mean HbO value during the baseline stage.

To examine the associations between cardiovascular changes and cortical hemodynamic responses, Spearman rank correlation analysis were performed between late-stage BP/HR reductions and late-stage ROI-level ΔHbO. Late-stage ΔSBP, ΔDBP, and ΔHR were calculated as the value at 0 min minus the value at 20 min, whereas late-stage ΔHbO was calculated as the mean HbO value during 15–20 min minus the mean baseline HbO value. False discovery rate (FDR) correction was applied to account for multiple correlation tests. A sensitivity analysis restricted to the three stimulation groups was also conducted after excluding the resting control group.

All randomized participants completed the intervention, and no outcome data were missing. All participants were analyzed according to their assigned groups. All statistical tests were two-sided, and P < 0.05 was considered statistically significant.

## 3 Results

### 3.1 Blood pressure and heart rate responses

Changes in SBP, DBP, and HR are shown in **Table 2**. Mixed-design repeated-measures ANOVA showed significant time effects for SBP, DBP, and HR (all P < 0.001), as well as significant group × time interactions for SBP (P < 0.001), DBP (P = 0.011), and HR (P = 0.037). No significant group effects were observed for these variables. Bonferroni-corrected within-group comparisons showed significant late-stage reductions in SBP across the three stimulation groups. Significant DBP reductions were mainly observed in the moxibustion aroma and combined stimulation groups, whereas HR reductions were mainly observed in the lower-limb warm and combined stimulation groups.

**Table 2:** Changes in SBP, DBP, and HR at different time points in the four groups and results of repeated-measures ANOVA.

Variable	Time (min)	Resting control (n = 10)	Moxibustion aroma stimulation (n = 10)	Lower-limb warm stimulation (n = 10)	Combined stimulation (n = 10)	Group effect (P)	Time effect (P)	Group × Time (P)
SBP (mmHg)	0	116.4 ± 3.9	119.7 ± 5.0	116.4 ± 7.6	118.0 ± 6.2	0.102	<0.001	<0.001
	5	117.5 ± 3.9	116.7 ± 5.5	112.8 ± 10.4	114.3 ± 6.2^a^			
	10	116.0 ± 3.7	113.5 ± 6.7^a^	108.8 ± 8.2^a^	112.9 ± 8.0^a^			
	15	116.9 ± 4.3	113.5 ± 8.2^a^	106.9 ± 8.1^a^	111.0 ± 6.5^a^			
	20	116.5 ± 3.6	109.6 ± 9.8^a^	103.8 ± 7.8^a^	108.6 ± 8.3^a^			
DBP (mmHg)	0	74.5 ± 3.4	79.3 ± 6.5	77.9 ± 8.3	76.4 ± 6.7	0.843	<0.001	0.011
	5	75.2 ± 3.1	77.2 ± 6.4	75.5 ± 8.8	75.4 ± 7.4			
	10	74.1 ± 3.3	75.7 ± 5.0	74.9 ± 8.5	74.6 ± 7.4			
	15	75.0 ± 3.7	75.1 ± 6.0^a^	73.7 ± 8.5	72.2 ± 8.1^a^			
	20	74.7 ± 3.3	73.4 ± 6.3	72.8 ± 9.3	69.5 ± 7.8^a^			
HR (beats/min)	0	72.0 ± 3.7	79.3 ± 9.0	77.4 ± 14.0	75.9 ± 13.0	0.335	<0.001	0.037
	5	71.8 ± 3.3	81.0 ± 10.2	76.0 ± 12.9	73.2 ± 9.4			
	10	72.2 ± 4.2	78.8 ± 12.0	74.5 ± 10.7	72.6 ± 8.7			
	15	72.0 ± 3.7	76.4 ± 8.1	72.6 ± 11.7^a^	69.6 ± 9.1^a^			
	20	71.8 ± 3.3	78.0 ± 10.4	71.5 ± 13.2^a^	68.9 ± 10.3^a^			

Values are presented as mean ± SD. ^a^P < 0.05 vs. 0 min within the same group after Bonferroni correction. SBP, systolic blood pressure; DBP, diastolic blood pressure; HR, heart rate.

**Figure 3:**
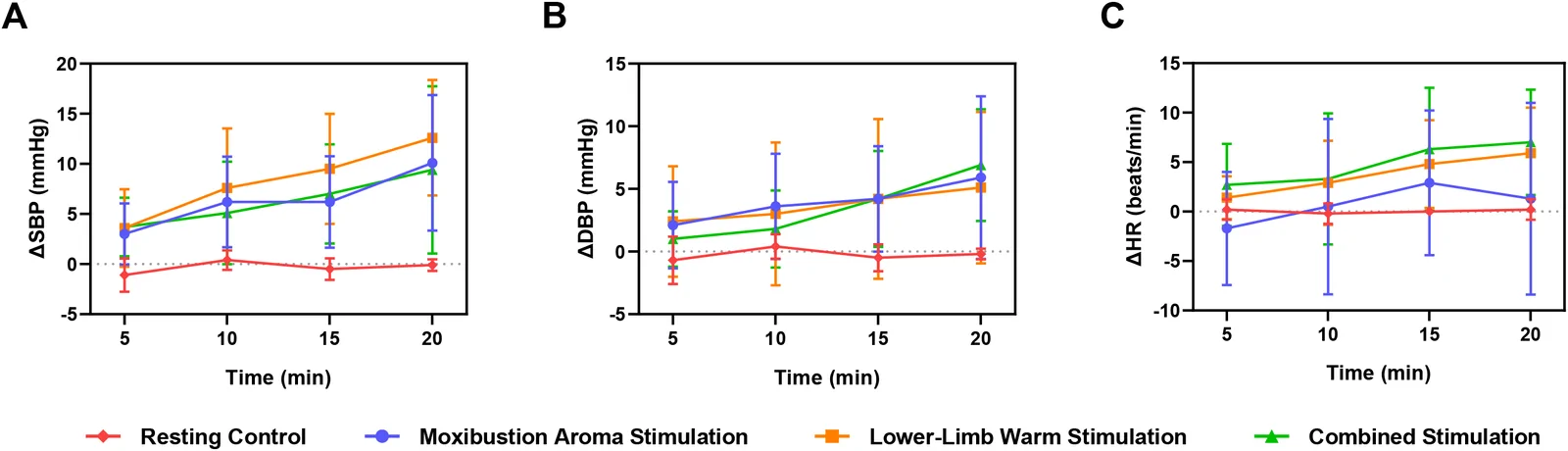
Reductions in SBP, DBP, and HR from baseline. Reductions were calculated as the value at 0 min minus the value at each subsequent time point. Positive values indicate reductions from baseline. Data are presented as mean ± SD. **(A)** reduction in systolic blood pressure (ΔSBP); **(B)** reduction in diastolic blood pressure (ΔDBP); **(C)** reduction in heart rate (ΔHR).

Reductions from 0 min in SBP, DBP, and HR are shown in **Figure 3**. Compared with the resting control group, the stimulation groups showed greater reductions at selected time points, particularly for ΔSBP. Significant differences in ΔDBP and ΔHR were mainly observed at later time points. Detailed analysis of reductions from 0 min and Bonferroni-corrected *post hoc* comparisons are provided in **Supplementary Tables 2** and **3**.

### 3.2 ROI-level HbO responses

ROI-level HbO responses are shown in **Table 3**. For LPFC HbO, significant group, stage, and group × stage effects were observed (all P < 0.001). For RPFC and LMC HbO, significant stage effects and group × stage interactions were observed (all P < 0.001), whereas the group effects were not significant. For RMC HbO, significant group and group × stage effects were observed (both P < 0.001), while the stage effect was not significant (P = 0.060). Bonferroni-corrected within-group comparisons showed significant changes from baseline mainly in the stimulation groups. LPFC HbO increased significantly at both intervention stages in all three stimulation groups. Significant RPFC, LMC, and RMC changes were observed in selected stimulation groups and stages, whereas no significant baseline-related changes were observed in the resting control group.

**Table 3:** Changes in ROI-level HbO at different stages in the four groups and results of repeated-measures ANOVA.

ROI	Stages (min)	Resting control (n = 10)	Moxibustion aroma stimulation (n = 10)	Lower-limb warm stimulation (n = 10)	Combined stimulation (n = 10)	Group effect (P)	Stage effect (P)	Group × Stage (P)
LPFC	Baseline	0.00 ± 0.09	0.03 ± 0.14	-0.18 ± 0.18	-0.38 ± 0.14	<0.001	<0.001	<0.001
	0–5	-0.02 ± 0.12	0.14 ± 0.11^a^	0.12 ± 0.10^a^	0.05 ± 0.17^a^			
	15–20	0.04 ± 0.08	0.18 ± 0.12^a^	0.24 ± 0.29^a^	0.06 ± 0.22^a^			
RPFC	Baseline	0.04 ± 0.08	0.02 ± 0.19	-0.10 ± 0.11	-0.05 ± 0.26	0.215	<0.001	<0.001
	0–5	0.02 ± 0.19	0.06 ± 0.13^a^	0.08 ± 0.11^a^	0.09 ± 0.34^a^			
	15–20	0.04 ± 0.23	-0.02 ± 0.11	0.24 ± 0.18^a^	0.07 ± 0.05^a^			
LMC	Baseline	0.01 ± 0.10	0.02 ± 0.26	-0.05 ± 0.16	-0.10 ± 0.27	0.083	<0.001	<0.001
	0–5	0.05 ± 0.23	0.14 ± 0.24^a^	-0.03 ± 0.22	-0.01 ± 0.38			
	15–20	0.03 ± 0.21	0.18 ± 0.28^a^	0.08 ± 0.18^a^	0.32 ± 0.22^a^			
RMC	Baseline	-0.04 ± 0.10	-0.04 ± 0.18	-0.07 ± 0.25	0.19 ± 0.41	<0.001	0.060	<0.001
	0–5	-0.02 ± 0.18	-0.20 ± 0.46^a^	0.25 ± 0.24^a^	0.17 ± 0.33			
	15–20	0.02 ± 0.16	0.04 ± 0.12^a^	0.09 ± 0.36^a^	0.10 ± 0.20^a^			

Values are presented as mean ± SD. ^a^P < 0.05 vs. baseline within the same group after Bonferroni correction. HbO, oxygenated hemoglobin; LPFC, left prefrontal cortex; RPFC, right prefrontal cortex; LMC, left motor cortex; RMC, right motor cortex.

**Figure 4:**
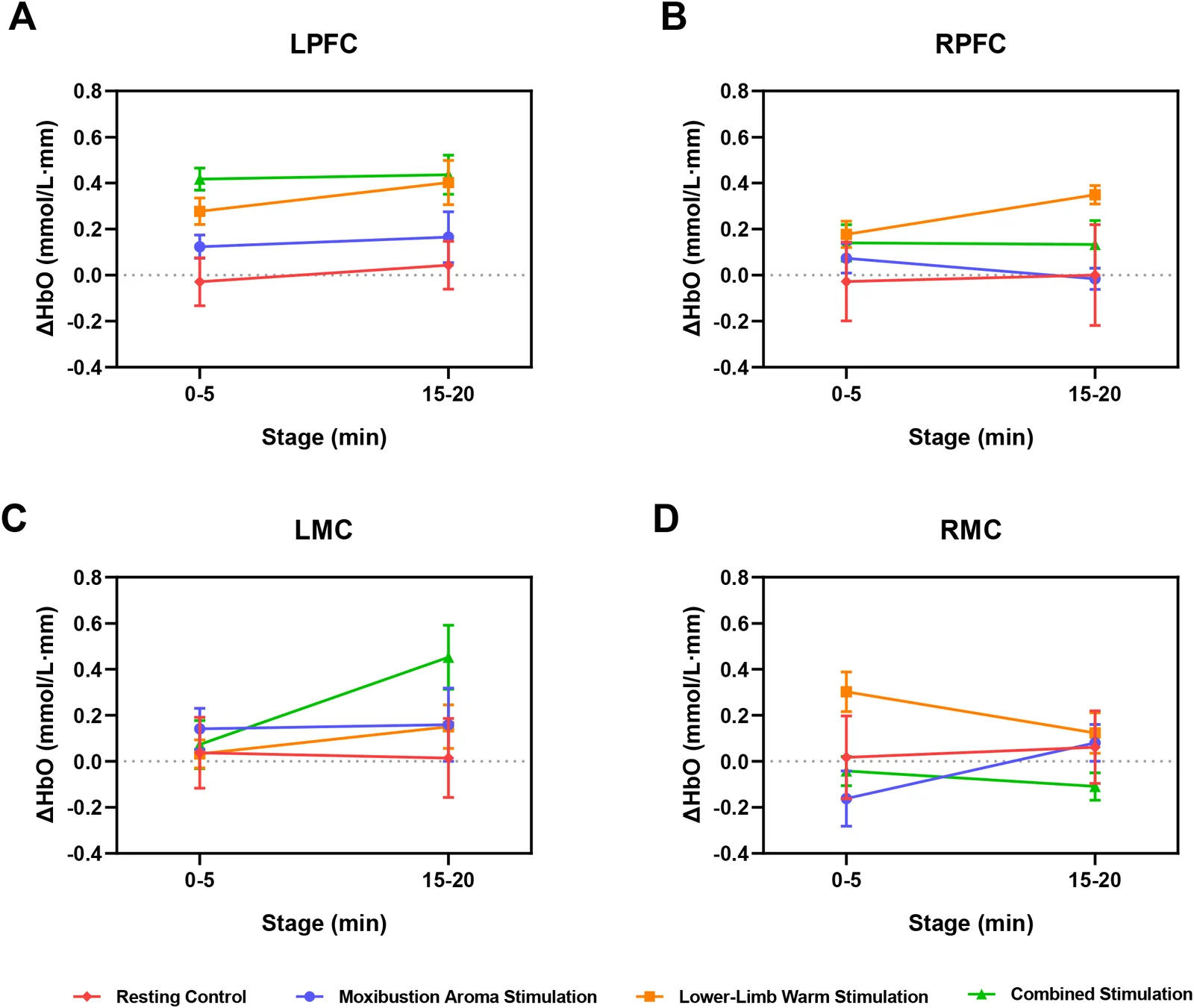
Changes in ROI-level HbO from baseline. ΔHbO was calculated as the value at each intervention stage minus the baseline value. Positive values indicate increases in HbO from baseline. Data are presented as mean ± SD. **(A)** LPFC; **(B)** RPFC; **(C)** LMC; **(D)** RMC.

Changes in ΔHbO from baseline are shown in **Figure 4**. The ΔHbO curves showed relatively small changes in the resting control group, whereas the stimulation groups exhibited ROI-specific increases, particularly in LPFC, RPFC, and LMC during the intervention period. Detailed analysis of ΔHbO and comparisons between each stimulation group and the resting control group are provided in **Supplementary Tables 4** and **5**.

### 3.3 Functional connectivity results

ROI-level FC results are shown in **Table 4**. Significant group × stage interactions were observed for all ROI-to-ROI connections, indicating that the temporal patterns of FC changes differed among the four groups. Significant group effects were observed for most ROI pairs, including LPFC–RPFC, LPFC–LMC, LPFC–RMC, RPFC–RMC, and LMC–RMC. Significant stage effects were observed for LPFC–RPFC, LPFC–LMC, and RPFC–LMC. Bonferroni-corrected within-group comparisons showed stage-related increases in FC across several ROI pairs, mainly in the stimulation groups. These increases were observed across different stimulation conditions, particularly during the late intervention stage, suggesting ROI-pair-specific network modulation rather than a response restricted to a single stimulation condition.

**Table 4:** Changes in ROI-level functional connectivity at different stages in the four groups and results of repeated-measures ANOVA.

ROI pair	Stages (min)	Resting control (n = 10)	Moxibustion aroma stimulation (n = 10)	Lower-limb warm stimulation (n = 10)	Combined stimulation (n = 10)	Group effect (P)	Stage effect (P)	Group × Stage (P)
LPFC–RPFC	Baseline	0.32 ± 0.06	0.32 ± 0.10	0.30 ± 0.11	0.31 ± 0.10	<0.001	<0.001	<0.001
	0–5	0.36 ± 0.10	0.45 ± 0.12^a^	0.36 ± 0.10	0.51 ± 0.11^a^			
	15–20	0.39 ± 0.13^a^	0.48 ± 0.13^a^	0.39 ± 0.11^a^	0.62 ± 0.12^a^			
LPFC–LMC	Baseline	0.15 ± 0.05	0.15 ± 0.09	0.16 ± 0.08	0.16 ± 0.09	<0.001	<0.001	<0.001
	0–5	0.16 ± 0.02	0.20 ± 0.10	0.22 ± 0.09^a^	0.30 ± 0.10^a^			
	15–20	0.21 ± 0.02	0.24 ± 0.11^a^	0.25 ± 0.10^a^	0.44 ± 0.12^a^			
LPFC–RMC	Baseline	0.18 ± 0.06	0.18 ± 0.08	0.15 ± 0.09	0.14 ± 0.08	<0.001	0.060	<0.001
	0–5	0.27 ± 0.10^a^	0.26 ± 0.10^a^	0.20 ± 0.10	0.31 ± 0.11^a^			
	15–20	0.28 ± 0.09^a^	0.30 ± 0.11^a^	0.23 ± 0.10^a^	0.47 ± 0.13^a^			
RPFC–LMC	Baseline	0.18 ± 0.08	0.17 ± 0.09	0.14 ± 0.08	0.15 ± 0.08	0.122	<0.001	<0.001
	0–5	0.20 ± 0.07	0.22 ± 0.10	0.19 ± 0.09	0.29 ± 0.10^a^			
	15–20	0.25 ± 0.07^a^	0.25 ± 0.11^a^	0.21 ± 0.09^a^	0.42 ± 0.12^a^			
RPFC–RMC	Baseline	0.23 ± 0.09	0.16 ± 0.08	0.16 ± 0.08	0.18 ± 0.09	<0.001	0.060	<0.001
	0–5	0.23 ± 0.09	0.20 ± 0.09	0.27 ± 0.10^a^	0.34 ± 0.11^a^			
	15–20	0.25 ± 0.06	0.23 ± 0.10^a^	0.31 ± 0.11^a^	0.49 ± 0.13^a^			
LMC–RMC	Baseline	0.25 ± 0.06	0.28 ± 0.11	0.29 ± 0.10	0.28 ± 0.11	<0.001	0.060	<0.001
	0–5	0.27 ± 0.11	0.30 ± 0.11	0.47 ± 0.12^a^	0.46 ± 0.12^a^			
	15–20	0.32 ± 0.11^a^	0.33 ± 0.12^a^	0.52 ± 0.13^a^	0.58 ± 0.14^a^			

Values are presented as mean ± SD. ^a^P < 0.05 vs. baseline within the same group after Bonferroni correction. FC, functional connectivity; LPFC, left prefrontal cortex; RPFC, right prefrontal cortex; LMC, left motor cortex; RMC, right motor cortex.

### 3.4 Associations between BP and HR and cortical hemodynamic responses

**Figure 5:**
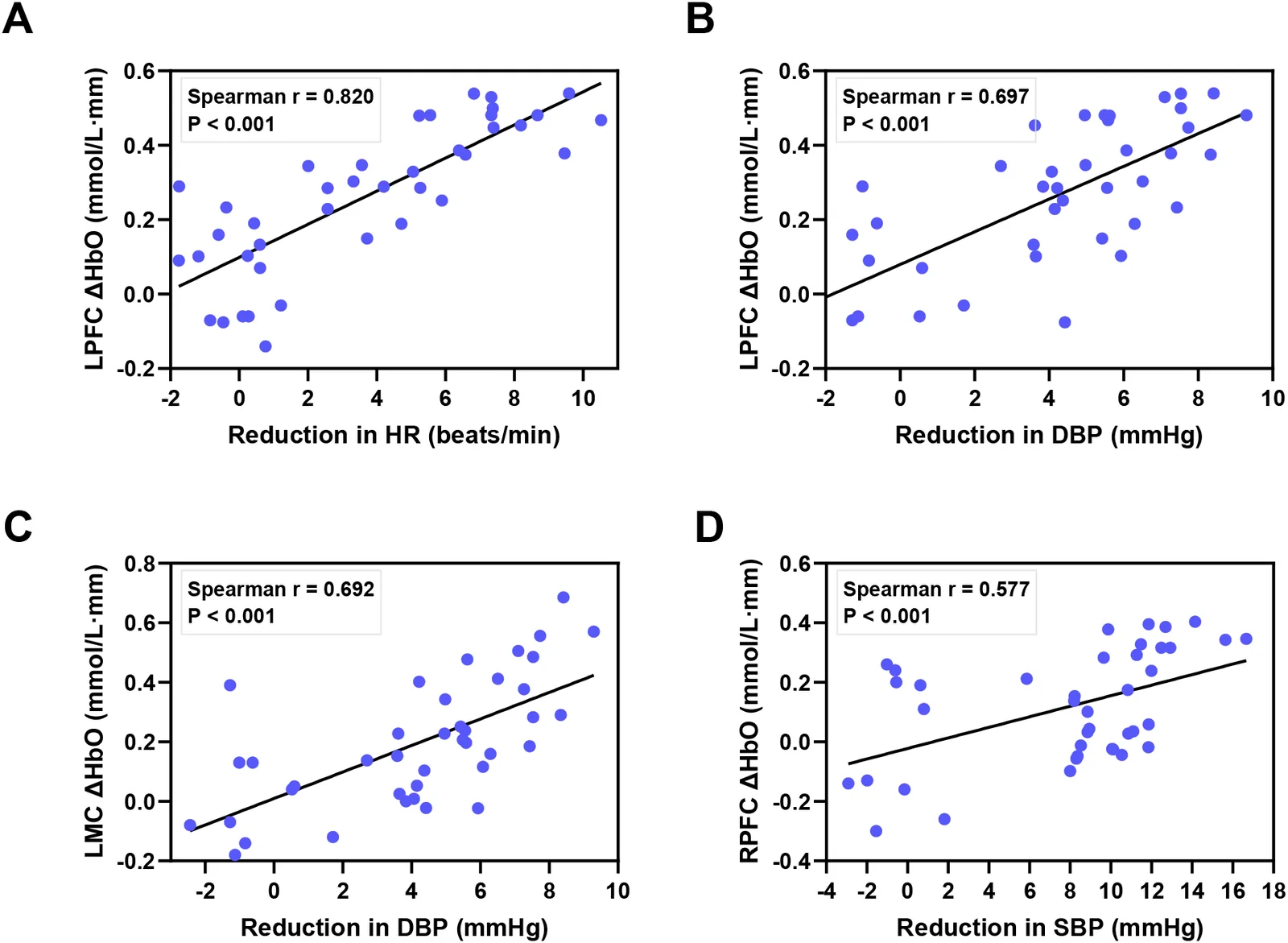
Correlations between late-stage SBP, DBP, HR reductions and ROI-level HbO changes. Late-stage SBP, DBP, and HR reductions were calculated as the value at 0 min minus the value at 20 min. Late-stage ΔHbO was calculated as the HbO value during 15–20 min minus the baseline value. The fitted line is shown for visualization only. **(A)** correlation between HR reduction and LPFC ΔHbO; **(B)** correlation between DBP reduction and LPFC ΔHbO; **(C)** correlation between DBP reduction and LMC ΔHbO; **(D)** correlation between SBP reduction and RPFC ΔHbO.

Associations between late-stage reductions in BP and HR and cortical hemodynamic responses are shown in **Figure 5**. Spearman correlation analysis showed that a greater HR reduction was positively associated with a larger LPFC ΔHbO (r = 0.820, P < 0.001). A greater DBP reduction was positively associated with larger ΔHbO in the LPFC (r = 0.697, P < 0.001) and LMC (r = 0.692, P < 0.001). A greater SBP reduction was positively associated with a larger RPFC ΔHbO (r = 0.577, P < 0.001).

Complete correlation results with FDR-adjusted q values are provided in **Supplementary Table 6**. A sensitivity analysis restricted to the three stimulation groups showed that several associations remained significant after FDR correction (**Supplementary Table 7**). These findings suggest that reductions in BP and HR were associated with ROI-specific increases in cortical HbO, particularly in prefrontal and motor-related regions.

## 4 Discussion

The present study investigated the acute effects of moxibustion aroma stimulation, lower-limb warm stimulation, and their combination on cardiovascular and cortical hemodynamic responses in healthy young adult men. The three stimulation conditions produced distinct temporal patterns of BP, HR, regional HbO, and HbO-based FC. In addition, greater late-stage reductions in cardiovascular measures were associated with larger HbO changes in selected cortical regions. Collectively, these findings suggest that the stimulation conditions elicited time-dependent cardiovascular responses accompanied by region- and stage-specific cortical hemodynamic changes.

For cardiovascular responses, significant time effects and group × time interactions were observed for the raw SBP, DBP, and HR values, whereas the overall group effects were not significant. The resting control group remained relatively stable, whereas SBP generally decreased over time in all three stimulation groups. Significant within-group reductions in DBP were mainly observed in the moxibustion aroma and combined stimulation groups, whereas significant HR reductions were mainly observed in the lower-limb warm and combined stimulation groups at later time points. Analyses of reductions from the pre-intervention level further showed that, compared with the resting control group, one or more stimulation groups exhibited greater reductions at selected time points, particularly for ΔSBP; however, these differences were not consistently observed across all stimulation groups or time points. The cardiovascular responses associated with lower-limb warm stimulation may reflect peripheral vasodilation, increased lower-limb blood flow, and consequent changes in peripheral vascular resistance and blood-volume distribution (Brunt et al., 2016b; Thomas et al., 2017; Cheng et al., 2021; Coates et al., 2026). Moxibustion aroma stimulation may be associated with cardiovascular responses through olfactory-related pathways, whereas the combined intervention may involve both thermal/somatosensory and olfactory inputs (Soudry et al., 2011; Thompson and Umphred, 2019; Cheng et al., 2021; Menzies et al., 2025; Toya et al., 2026). However, these possible pathways remain speculative because autonomic involvement was not directly assessed and direct autonomic and vascular measures were not collected. Overall, the four groups exhibited different temporal cardiovascular response profiles; nevertheless, a significant within-group change in one condition but not another does not itself demonstrate a significant between-group difference or the superiority of any intervention.

The fNIRS results showed ROI-, stage-, and stimulation-specific cortical hemodynamic responses, with significant group × stage interactions observed in all four ROIs. LPFC HbO increased from baseline during both intervention stages in all three stimulation groups, indicating that increased LPFC HbO was a common directional response across the stimulation conditions, whereas changes in RPFC, LMC, and RMC HbO occurred only in selected groups and stages. This common LPFC response is consistent with previous evidence linking prefrontal regions to autonomic and cardiovascular regulation (Thayer et al., 2012; Shantsila, 2016); however, the present findings should not be interpreted as direct evidence of autonomic modulation. Baseline-corrected ΔHbO comparisons further showed that differences between the stimulation and resting control groups were specific to the ROI, stimulation condition, and intervention stage. Because the overall group effects for RPFC and LMC HbO were not significant, isolated stage-specific differences in these regions should not be generalized as consistent overall between-group differences. HbO-based FC analysis also revealed significant group × stage interactions across all ROI pairs, with increases in several intra-prefrontal and fronto-motor connections occurring mainly in the stimulation groups and more frequently during the late intervention stage. These findings indicate coordinated interregional hemodynamic fluctuations; however, because FC was derived from correlations between regional HbO signals, it should not be interpreted as direct evidence of altered neuronal connectivity.

The correlation analysis further showed associations between peripheral cardiovascular responses and cortical hemodynamic changes. A greater HR reduction was associated with a larger LPFC ΔHbO; a greater DBP reduction was associated with larger ΔHbO in the LPFC and LMC; and a greater SBP reduction was associated with a larger RPFC ΔHbO. Several of these associations remained significant after FDR correction in the sensitivity analysis restricted to the three stimulation groups, suggesting that they were not solely driven by overall differences between the resting control and stimulation groups. The region-specific pattern indicates that different cardiovascular responses covaried with hemodynamic changes in different cortical regions. Nevertheless, these findings represent associations rather than evidence of a direct central–peripheral regulatory pathway. Because cardiovascular and HbO changes were measured over the same intervention period, the direction of these relationships cannot be determined. Cortical hemodynamic changes may have accompanied cardiovascular regulation, systemic cardiovascular changes may have influenced the fNIRS signals, or both may have reflected shared physiological responses to stimulation.

Several limitations should be acknowledged. First, the sample size was small, with only 10 participants in each group, and no formal *a priori* sample-size calculation was performed; therefore, the study may have been underpowered to detect small or moderate between-group differences. Second, only healthy young adult men were included. Therefore, the present findings should not be generalized to women, older adults, or patients with hypertension until confirmed in larger, appropriately powered clinical studies involving these populations. Third, only acute responses during a single 20-min session were assessed, without post-intervention or long-term follow-up. Fourth, the study was not prospectively registered and no blinding was implemented, which may have increased the risks of reporting, performance, and assessment bias. Fifth, the resting control condition helped control for time-related changes during quiet sitting but did not control for nonspecific effects such as attention, expectation, sensory exposure, or participant–investigator interaction. Therefore, differences between the stimulation and resting control groups cannot be attributed solely to the specific olfactory or thermal components of the interventions. Sixth, direct autonomic and vascular indices, including HR variability, baroreflex sensitivity, respiration, skin conductance, peripheral skin temperature, and vascular resistance, were not measured. Finally, fNIRS primarily measures superficial cortical hemodynamic signals and cannot directly assess deeper autonomic structures or establish neural causality. Future multicenter studies with larger sample sizes, prospective trial registration, blinded outcome assessment where feasible, appropriately matched sham or sensory/attention control conditions, direct autonomic and vascular measurements, and longer follow-up are needed to confirm these preliminary findings and better clarify their physiological mechanisms and clinical relevance.

## 5 Conclusion

This pilot randomized trial showed that moxibustion aroma stimulation, lower-limb warm stimulation, and combined stimulation were associated with distinct time-dependent patterns of BP, HR, and cortical hemodynamic responses in healthy young adult men. The cardiovascular variables showed significant group × time interactions, whereas fNIRS demonstrated ROI- and stage-specific HbO responses and changes in HbO-based interregional coupling. Greater late-stage reductions in BP and HR were associated with larger increases in HbO in selected cortical regions. These findings support an association between acute peripheral cardiovascular responses and cortical hemodynamic changes during stimulation. However, they do not establish a causal central–peripheral regulatory mechanism or demonstrate the superiority of any intervention. Larger multicenter studies with prospective registration, blinded outcome assessment where feasible, and direct autonomic and vascular measurements are warranted to confirm these preliminary findings.

## Data Availability

The original contributions presented in the study are included in the article/**Supplementary Material**. Further inquiries can be directed to the corresponding author.
