# Supplementary material for: Effects of moxibustion aroma stimulation and lower-limb warm stimulation on blood pressure and cerebral hemodynamics: a pilot randomized trial
Source: Front Physiol. 2026 Sep 8;17:1898938. doi: 10.3389/fphys.2026.1898938 (PMC13593865; doi:10.3389/fphys.2026.1898938)
Supplement: Supplementary file 1 [file SupplementaryFile1.docx]

Supplementary Material

**Supplementary Table 1. CONSORT 2025 checklist of information to include when reporting a randomised trial**

| **Section / Topic** | **No** | **CONSORT 2025 checklist item description** | **Reported on page no.** |
| --- | --- | --- | --- |
| **Title and abstract** | | |  |
| Title and structured abstract | 1a | Identification as a randomised trial | 1 |
|  | 1b | Structured summary of the trial design, methods, results, and conclusions | 1 |
| **Open science** | | |  |
| Trial registration | 2 | Name of trial registry, identifying number (with URL) and date of registration | 3 |
| Protocol and statistical analysis plan | 3 | Where the trial protocol and statistical analysis plan can be accessed | Not Applicable |
| Data sharing | 4 | Where and how the individual de-identified participant data (including data dictionary), statistical code and any other materials can be accessed | Not reported |
| Funding and conflicts of interest | 5a | Sources of funding and other support (e.g., supply of drugs), and role of funders in the design, conduct, analysis and reporting of the trial | 11 |
|  | 5b | Financial and other conflicts of interest of the manuscript authors | 10 |
| **Introduction** | | |  |
| Background and rationale | 6 | Scientific background and rationale | 2 |
| Objectives | 7 | Specific objectives related to benefits and harms | 3 |
| **Methods** | | |  |
| Patient and public involvement | 8 | Details of patient or public involvement in the design, conduct and reporting of the trial | Not reported |
| Trial design | 9 | Description of trial design including type of trial (e.g., parallel group, crossover), allocation ratio, and framework (e.g., superiority, equivalence, non-inferiority, exploratory) | 3-4 |
| Changes to trial protocol | 10 | Important changes to the trial after it commenced including any outcomes or analyses that were not prespecified, with reason | Not applicable |
| Trial setting | 11 | Settings (e.g., community, hospital) and locations (e.g., countries, sites) where the trial was conducted | 3 |
| Eligibility criteria | 12a | Eligibility criteria for participants | 3-4 |
|  | 12b | If applicable, eligibility criteria for sites and for individuals delivering the interventions (e.g., surgeons, physiotherapists) | Not Applicable |
| Intervention and comparator | 13 | Intervention and comparator with sufficient details to allow replication. If relevant, where additional materials describing the intervention and comparator (e.g., intervention manual) can be accessed | 4-5 |
| Outcomes | 14 | Pre-specified primary and secondary outcomes, including the specific measurement variable (e.g., systolic blood pressure), analysis metric (e.g., change from baseline, final value, time to event), method of aggregation (e.g., median, proportion), and time point for each outcome | 5-7 |
| Harms | 15 | How harms were defined and assessed (e.g., systematically, non-systematically) | Not reported |
| Sample size | 16a | How sample size was determined, including all assumptions supporting the sample size calculation | 4 |
|  | 16b | Explanation of any interim analyses and stopping guidelines | Not Applicable |
| Randomisation: |  |  |  |
| Sequence generation | 17a | Who generated the random allocation sequence and the method used | 4 |
|  | 17b | Type of randomisation and details of any restriction (e.g., stratification, blocking and block size) | 4 |
| Allocation concealment mechanism | 18 | Mechanism used to implement the random allocation sequence (e.g., central computer/telephone; sequentially numbered, opaque, sealed containers), describing any steps to conceal the sequence until interventions were assigned | 4 |
| Implementation | 19 | Whether the personnel who enrolled and those who assigned participants to the interventions had access to the random allocation sequence | 4 |
| Blinding | 20a | Who was blinded after assignment to interventions (e.g., participants, care providers, outcome assessors, data analysts) | 4 |
|  | 20b | If blinded, how blinding was achieved and description of the similarity of interventions | Not Applicable |
| Statistical methods | 21a | Statistical methods used to compare groups for primary and secondary outcomes, including harms | 6-7 |
|  | 21b | Definition of who is included in each analysis (e.g., all randomised participants), and in which group | 7 |
|  | 21c | How missing data were handled in the analysis | 7 |
|  | 21d | Methods for any additional analyses (e.g., subgroup and sensitivity analyses), distinguishing prespecified from post-hoc | 7 |
| **Results** | | |  |
| Participant flow, including flow diagram | 22a | For each group, the numbers of participants who were randomly assigned, received intended intervention, and were analysed for the primary outcome | 1, 7, 15-18 |
|  | 22b | For each group, losses and exclusions after randomisation, together with reasons | 7 |
| Recruitment | 23a | Dates defining the periods of recruitment and follow-up for outcomes of benefits and harms | 3 |
|  | 23b | If relevant, why the trial ended or was stopped | Not Applicable |
| Intervention and comparator delivery | 24a | Intervention and comparator as they were actually administered (e.g., where appropriate, who delivered the intervention/comparator, how participants adhered, whether they were delivered as intended [fidelity]) | 4-5, 7 |
|  | 24b | Concomitant care received during the trial for each group | Not Applicable |
| Baseline data | 25 | A table showing baseline demographic and clinical characteristics for each group | 15 |
| Numbers analysed,  outcomes and estimation | 26 | For each primary and secondary outcome, by group:   - the number of participants included in the analysis - the number of participants with available data at the outcome time point - result for each group, and the estimated effect size and its precision (such as 95% confidence interval) - for binary outcomes, presentation of both absolute and relative effect size | 7-8, 16-18, 21-23; Suppl. Tables 2-7 |
| Harms | 27 | All harms or unintended events in each group | Not reported |
| Ancillary analyses | 28 | Any other analyses performed, including subgroup and sensitivity analyses, distinguishing pre-specified from post-hoc | 7-8; Suppl. Table 7 |
| **Discussion** | | |  |
| Interpretation | 29 | Interpretation consistent with results, balancing benefits and harms, and considering other relevant evidence | 8-10 |
| Limitations | 30 | Trial limitations, addressing sources of potential bias, imprecision, generalisability, and, if relevant, multiplicity of analyses | 10 |

**Supplementary Table 2.** Mixed-design repeated-measures ANOVA results for reductions from baseline in SBP, DBP, and HR.

| **Outcome** | **Effect** | **F** | **P** | **Partial eta squared** |
| --- | --- | --- | --- | --- |
| ΔSBP | Group | 11.49 | **<0.001** | 0.489 |
| ΔSBP | Time | 18.30 | **<0.001** | 0.337 |
| ΔSBP | Group × Time | 1.97 | 0.050 | 0.141 |
| ΔDBP | Group | 3.31 | **0.031** | 0.216 |
| ΔDBP | Time | 10.78 | **<0.001** | 0.230 |
| ΔDBP | Group × Time | 1.83 | 0.071 | 0.132 |
| ΔHR | Group | 2.40 | 0.084 | 0.167 |
| ΔHR | Time | 10.23 | **<0.001** | 0.221 |
| ΔHR | Group × Time | 1.57 | 0.133 | 0.116 |

Note: Δ values were calculated as the value at 0 min minus the value at each subsequent time point; positive values indicate reductions from baseline. Time points included in this analysis were 5, 10, 15, and 20 min. Partial eta squared is reported as the effect size. P values < 0.05 were considered statistically significant; the P = 0.050 result is reported for transparency and should be interpreted cautiously. SBP, systolic blood pressure; DBP, diastolic blood pressure; HR, heart rate.

**Supplementary Table 3.** Bonferroni-corrected post hoc comparisons between stimulation groups and the resting control group for SBP, DBP, and HR.

| **Outcome** | **Time (min)** | **Comparison** | **Unadjusted P** | **Bonferroni-adjusted P** |
| --- | --- | --- | --- | --- |
| ΔSBP | 5 | Combined Stimulation vs Resting Control | <0.001 | **0.005** |
| ΔSBP | 5 | Moxibustion Aroma Stimulation vs Resting Control | 0.002 | **0.027** |
| ΔSBP | 5 | Lower-Limb Warm Stimulation vs Resting Control | 0.004 | **0.048** |
| ΔSBP | 10 | Combined Stimulation vs Resting Control | 0.018 | 0.211 |
| ΔSBP | 10 | Moxibustion Aroma Stimulation vs Resting Control | 0.003 | **0.033** |
| ΔSBP | 10 | Lower-Limb Warm Stimulation vs Resting Control | 0.004 | **0.047** |
| ΔSBP | 15 | Combined Stimulation vs Resting Control | <0.001 | **0.011** |
| ΔSBP | 15 | Moxibustion Aroma Stimulation vs Resting Control | 0.001 | **0.013** |
| ΔSBP | 15 | Lower-Limb Warm Stimulation vs Resting Control | <0.001 | **0.003** |
| ΔSBP | 20 | Combined Stimulation vs Resting Control | 0.006 | 0.069 |
| ΔSBP | 20 | Moxibustion Aroma Stimulation vs Resting Control | <0.001 | **0.012** |
| ΔSBP | 20 | Lower-Limb Warm Stimulation vs Resting Control | <0.001 | **<0.001** |
| ΔDBP | 5 | Combined Stimulation vs Resting Control | 0.081 | 0.977 |
| ΔDBP | 5 | Moxibustion Aroma Stimulation vs Resting Control | 0.041 | 0.490 |
| ΔDBP | 5 | Lower-Limb Warm Stimulation vs Resting Control | 0.063 | 0.754 |
| ΔDBP | 10 | Combined Stimulation vs Resting Control | 0.199 | 1.000 |
| ΔDBP | 10 | Moxibustion Aroma Stimulation vs Resting Control | 0.041 | 0.489 |
| ΔDBP | 10 | Lower-Limb Warm Stimulation vs Resting Control | 0.187 | 1.000 |
| ΔDBP | 15 | Combined Stimulation vs Resting Control | 0.004 | **0.043** |
| ΔDBP | 15 | Moxibustion Aroma Stimulation vs Resting Control | 0.006 | 0.077 |
| ΔDBP | 15 | Lower-Limb Warm Stimulation vs Resting Control | 0.046 | 0.547 |
| ΔDBP | 20 | Combined Stimulation vs Resting Control | <0.001 | **0.008** |
| ΔDBP | 20 | Moxibustion Aroma Stimulation vs Resting Control | 0.016 | 0.188 |
| ΔDBP | 20 | Lower-Limb Warm Stimulation vs Resting Control | 0.022 | 0.261 |
| ΔHR | 5 | Combined Stimulation vs Resting Control | 0.093 | 1.000 |
| ΔHR | 5 | Moxibustion Aroma Stimulation vs Resting Control | 0.326 | 1.000 |
| ΔHR | 5 | Lower-Limb Warm Stimulation vs Resting Control | 0.139 | 1.000 |
| ΔHR | 10 | Combined Stimulation vs Resting Control | 0.131 | 1.000 |
| ΔHR | 10 | Moxibustion Aroma Stimulation vs Resting Control | 0.809 | 1.000 |
| ΔHR | 10 | Lower-Limb Warm Stimulation vs Resting Control | 0.049 | 0.587 |
| ΔHR | 15 | Combined Stimulation vs Resting Control | 0.011 | 0.127 |
| ΔHR | 15 | Moxibustion Aroma Stimulation vs Resting Control | 0.241 | 1.000 |
| ΔHR | 15 | Lower-Limb Warm Stimulation vs Resting Control | 0.008 | 0.092 |
| ΔHR | 20 | Combined Stimulation vs Resting Control | 0.003 | **0.034** |
| ΔHR | 20 | Moxibustion Aroma Stimulation vs Resting Control | 0.729 | 1.000 |
| ΔHR | 20 | Lower-Limb Warm Stimulation vs Resting Control | 0.003 | **0.041** |

Note: Pairwise comparisons were performed between each stimulation group and the resting control group at the same time point. Bold Bonferroni-adjusted P values indicate statistical significance at P < 0.05. SBP, systolic blood pressure; DBP, diastolic blood pressure; HR, heart rate.

**Supplementary Table 4.** Mixed-design repeated-measures ANOVA results for changes from baseline in ROI-level HbO.

| **Outcome** | **Effect** | **F** | **P** | **Partial eta squared** |
| --- | --- | --- | --- | --- |
| ΔHbO-LPFC | Group | 63.12 | **<0.001** | 0.840 |
| ΔHbO-LPFC | Stage | 15.50 | **<0.001** | 0.301 |
| ΔHbO-LPFC | Group × Stage | 1.95 | 0.139 | 0.140 |
| ΔHbO-RPFC | Group | 13.35 | **<0.001** | 0.527 |
| ΔHbO-RPFC | Stage | 3.92 | 0.055 | 0.098 |
| ΔHbO-RPFC | Group × Stage | 17.05 | **<0.001** | 0.587 |
| ΔHbO-LMC | Group | 7.67 | **<0.001** | 0.390 |
| ΔHbO-LMC | Stage | 49.84 | **<0.001** | 0.581 |
| ΔHbO-LMC | Group × Stage | 26.87 | **<0.001** | 0.691 |
| ΔHbO-RMC | Group | 15.78 | **<0.001** | 0.568 |
| ΔHbO-RMC | Stage | 0.47 | 0.499 | 0.013 |
| ΔHbO-RMC | Group × Stage | 37.03 | **<0.001** | 0.755 |

Note: ΔHbO values were calculated as the value at each intervention stage minus the baseline value; positive values indicate increases from baseline. Stages included in this analysis were the early intervention period (0–5 min) and late intervention period (15–20 min). Partial eta squared is reported as the effect size. P values < 0.05 were considered statistically significant. HbO, oxygenated hemoglobin; LPFC, left prefrontal cortex; RPFC, right prefrontal cortex; LMC, left motor cortex; RMC, right motor cortex.

**Supplementary Table 5.** Bonferroni-corrected post hoc comparisons between stimulation groups and the resting control group for ΔHbO.

| **ROI** | **Stage** | **Comparison** | **Unadjusted P** | **Bonferroni-adjusted P** |
| --- | --- | --- | --- | --- |
| LPFC | 0–5 | Moxibustion Aroma Stimulation vs Resting Control | 0.001 | **0.007** |
| LPFC | 0–5 | Lower-Limb Warm Stimulation vs Resting Control | <0.001 | **<0.001** |
| LPFC | 0–5 | Combined Stimulation vs Resting Control | <0.001 | **<0.001** |
| LPFC | 15–20 | Moxibustion Aroma Stimulation vs Resting Control | 0.045 | 0.269 |
| LPFC | 15–20 | Lower-Limb Warm Stimulation vs Resting Control | <0.001 | **<0.001** |
| LPFC | 15–20 | Combined Stimulation vs Resting Control | <0.001 | **<0.001** |
| RPFC | 0–5 | Moxibustion Aroma Stimulation vs Resting Control | 0.108 | 0.646 |
| RPFC | 0–5 | Lower-Limb Warm Stimulation vs Resting Control | 0.004 | **0.026** |
| RPFC | 0–5 | Combined Stimulation vs Resting Control | 0.015 | 0.091 |
| RPFC | 15–20 | Moxibustion Aroma Stimulation vs Resting Control | 0.821 | 1.000 |
| RPFC | 15–20 | Lower-Limb Warm Stimulation vs Resting Control | <0.001 | **0.004** |
| RPFC | 15–20 | Combined Stimulation vs Resting Control | 0.107 | 0.642 |
| LMC | 0–5 | Moxibustion Aroma Stimulation vs Resting Control | 0.086 | 0.517 |
| LMC | 0–5 | Lower-Limb Warm Stimulation vs Resting Control | 0.921 | 1.000 |
| LMC | 0–5 | Combined Stimulation vs Resting Control | 0.560 | 1.000 |
| LMC | 15–20 | Moxibustion Aroma Stimulation vs Resting Control | 0.066 | 0.399 |
| LMC | 15–20 | Lower-Limb Warm Stimulation vs Resting Control | 0.046 | 0.275 |
| LMC | 15–20 | Combined Stimulation vs Resting Control | <0.001 | **<0.001** |
| RMC | 0–5 | Moxibustion Aroma Stimulation vs Resting Control | 0.018 | 0.110 |
| RMC | 0–5 | Lower-Limb Warm Stimulation vs Resting Control | <0.001 | **0.004** |
| RMC | 0–5 | Combined Stimulation vs Resting Control | 0.342 | 1.000 |
| RMC | 15–20 | Moxibustion Aroma Stimulation vs Resting Control | 0.744 | 1.000 |
| RMC | 15–20 | Lower-Limb Warm Stimulation vs Resting Control | 0.304 | 1.000 |
| RMC | 15–20 | Combined Stimulation vs Resting Control | 0.008 | **0.049** |

Note: Pairwise comparisons were performed between each stimulation group and the resting control group at the same intervention stage. Bold Bonferroni-adjusted P values indicate statistical significance at P < 0.05. HbO, oxygenated hemoglobin; LPFC, left prefrontal cortex; RPFC, right prefrontal cortex; LMC, left motor cortex; RMC, right motor cortex.

**Supplementary Table 6.** Spearman correlation analysis between late-stage SBP, DBP, HR reductions and ROI-level HbO changes.

| **Reduction** | **ROI-level ΔHbO** | **Spearman r** | **P value** | **FDR-adjusted q value** |
| --- | --- | --- | --- | --- |
| ΔSBP | LPFC ΔHbO | 0.620 | <0.001 | **<0.001** |
| ΔSBP | RPFC ΔHbO | 0.577 | <0.001 | **<0.001** |
| ΔSBP | LMC ΔHbO | 0.275 | 0.0861 | 0.1147 |
| ΔSBP | RMC ΔHbO | 0.217 | 0.1777 | 0.2132 |
| ΔDBP | LPFC ΔHbO | 0.697 | <0.001 | **<0.001** |
| ΔDBP | RPFC ΔHbO | 0.207 | 0.1997 | 0.2178 |
| ΔDBP | LMC ΔHbO | 0.692 | <0.001 | **<0.001** |
| ΔDBP | RMC ΔHbO | -0.291 | 0.069 | 0.1034 |
| ΔHR | LPFC ΔHbO | 0.820 | <0.001 | **<0.001** |
| ΔHR | RPFC ΔHbO | 0.544 | <0.001 | **<0.001** |
| ΔHR | LMC ΔHbO | 0.642 | <0.001 | **<0.001** |
| ΔHR | RMC ΔHbO | -0.173 | 0.2853 | 0.2853 |

Note: Late-stage SBP, DBP, and HR reductions were calculated as the value at 0 min minus the value at 20 min. Late-stage ΔHbO was calculated as the HbO value during 15–20 min minus the baseline value. Positive values indicate greater SBP, DBP, and HR reductions or greater HbO increases from baseline. Bold FDR-adjusted q values indicate statistical significance at q < 0.05. SBP, systolic blood pressure; DBP, diastolic blood pressure; HR, heart rate; HbO, oxygenated hemoglobin; LPFC, left prefrontal cortex; RPFC, right prefrontal cortex; LMC, left motor cortex; RMC, right motor cortex.

**Supplementary Table 7.** Sensitivity analysis of Spearman correlations restricted to the three stimulation groups.

| **Reduction** | **ROI-level ΔHbO** | **Spearman r** | **P value** | **FDR-adjusted q value** |
| --- | --- | --- | --- | --- |
| ΔSBP | LPFC ΔHbO | 0.330 | 0.0749 | 0.0998 |
| ΔSBP | RPFC ΔHbO | 0.614 | <0.001 | **0.0012** |
| ΔSBP | LMC ΔHbO | -0.216 | 0.2526 | 0.3031 |
| ΔSBP | RMC ΔHbO | 0.455 | 0.0115 | **0.0198** |
| ΔDBP | LPFC ΔHbO | 0.505 | 0.0045 | **0.0089** |
| ΔDBP | RPFC ΔHbO | 0.001 | 0.9963 | 0.9963 |
| ΔDBP | LMC ΔHbO | 0.650 | <0.001 | **<0.001** |
| ΔDBP | RMC ΔHbO | -0.387 | 0.0345 | 0.0517 |
| ΔHR | LPFC ΔHbO | 0.816 | <0.001 | **<0.001** |
| ΔHR | RPFC ΔHbO | 0.554 | 0.0015 | **0.0036** |
| ΔHR | LMC ΔHbO | 0.588 | <0.001 | **0.0019** |
| ΔHR | RMC ΔHbO | -0.189 | 0.3175 | 0.3464 |

Note: This sensitivity analysis included only the moxibustion aroma stimulation, lower-limb warm stimulation, and combined stimulation groups (n = 30). Late-stage SBP, DBP, HR reductions and ΔHbO were calculated using the same definitions as in Supplementary Table 6. Bold FDR-adjusted q values indicate statistical significance at q < 0.05. SBP, systolic blood pressure; DBP, diastolic blood pressure; HR, heart rate; HbO, oxygenated hemoglobin; LPFC, left prefrontal cortex; RPFC, right prefrontal cortex; LMC, left motor cortex; RMC, right motor cortex.
